# Trained Immunity Generated by the Recombinant Zoster Vaccine

**DOI:** 10.21203/rs.3.rs-4607744/v1

**Published:** 2024-07-10

**Authors:** Adriana Weinberg, Michael Johnson, Megan Crotteau, Debashis Ghosh, Thao Vu, Myron J Levin

**Affiliations:** University of Colorado Denver | Anschutz Medical Campus; University of Colorado Denver; University of Colorado Denver; University of Colorado Denver; University of Colorado Denver; University of Colorado Denver

**Keywords:** trained immunity, zoster, zoster vaccines, recombinant zoster vaccine, adaptive NK cells, monocyte immunologic memory, older adults

## Abstract

Trained immunity may play a role in vaccine-induced protection against infections. We showed that the highly efficacious recombinant VZV-gE zoster vaccine (RZV) generated trained immunity in monocytes, natural killer (NK) cells, and dendritic cells (DCs) and that the less efficacious live zoster vaccine did not. RZV stimulated ex vivo gE-specific monocyte, DC and NK cell responses that did not correlate with CD4 + T-cell responses. These responses were also elicited in purified monocyte and NK cell cocultures stimulated with VZV-gE and persisted above prevaccination levels for ≥ 4 years post-RZV administration. RZV administration also increased ex vivo heterologous monocyte and NK cell responses to herpes simplex and cytomegalovirus antigens. ATAC-seq analysis and ex vivo TGFβ1 supplementation and inhibition experiments demonstrated that decreased *tgfβ1* transcription resulting from RZV-induced chromatin modifications may explain the development of monocyte trained immunity. The role of RZV-trained immunity in protection against herpes zoster and other infections should be further studied.

## Introduction

Herpes zoster (HZ) is a severe disease caused by the reactivation of latent varicella-zoster virus (VZV). Asymptomatic VZV reactivations are not uncommon, but viral replication is generally controlled by the host immune response. Clinical manifestations of HZ ensue when the immune system fails to contain the viral spread. HZ is most frequent in people with decreased cell-mediated immunity (CMI), including those with congenital immunodeficiencies, AIDS, iatrogenic immunosuppression, and older adults with immunosenescence^1–4^. Moreover, the risk of HZ is not increased by isolated antibody deficiency, nor is it mitigated by providing antibody supplementation. Thus, protection against HZ is deemed to be primarily mediated by CMI. Additional evidence links the incidence of HZ to a decrease in VZV-specific Th1 responses^5^. However, the exact mechanisms that limit viral spread during VZV reactivation have not been defined. In addition to adaptive immunity, protection against HZ may involve innate cell-mediated immunity^6–8^.

Innate immune cells are rapidly deployed as the first line of defense against pathogens. Moreover, innate immune cells are critical for the development of antigen-specific adaptive CMI and for establishing feedback mechanisms that allow adaptive CMI to boost innate immunity^9–15^. Recent evidence revealed that innate immune cells develop memory-like responses, also known as *trained immunity*^11,15–26^. Unlike conventional T and B cells, innate immune cells either lack antigen-specific receptors or have a limited capacity for recombination. Instead, the development of memory in innate immune cells relies on epigenetic modifications and clonal expansions ^26,27^. The best studied viral infection controlled by trained immunity is cytomegalovirus (CMV), which, like VZV, is a member of the Herpesviridae family that is defined by a life cycle consisting of acute infection, followed by latency and reactivation. CMV-specific adaptive NK cell clones, which recognize CMV peptides in the context of HLA class I E, have high in vitro cytolytic capacity against CMV-infected cells and confer in vivo protection in a murine CMV infection model and in human transplant recipients^27–31^. These adaptive NK cells have well-characterized epigenetic modifications underlying their CMV specificity^27,32,33^. VZV-specific adaptive NK cells have also been described^34^.

Another set of innate immune cells with CMV-specific activity are the Vδ2^−^ and Vγ9^−^ γδ T cells^35–37^. These cells undergo limited T-cell receptor (TCR) rearrangement, which confers CMV specificity and the ability to control CMV reactivations^35–38^. Monocytes and dendritic cells (DCs) can also develop trained immunity^26,39^. The role of monocytes in the control of viral infection has been insufficiently studied, but monocytes in the context of *M. tuberculosis* infection develop trained immunity that confers protection against active disease^40^. Monocyte immunologic memory is maintained through epigenetic modifications of hematopoietic stem and progenitor cells^41,42^.

Vaccines are available to protect against many infections, including HZ, but people at the highest risk of developing these infections tend to have poor responses to vaccines. This is exemplified by the Zoster Vaccine Live (ZVL), which confers 70% protection against HZ in adults 50 to 59 years of age, 64% in adults aged 60–69 years, 41% in adults aged 70–79 years and no protection in adults aged ≥ 80 years ^43–45^. Moreover, the protection conferred by ZVL completely wanes 10 years after vaccination. In contrast, a recombinant zoster vaccine (RZV) - containing the VZV glycoprotein E (gE) combined with the potent adjuvant ASO1B - changed this paradigm. RZV achieved 3-year efficacy > 90% and 10-year efficacy > 70% against zoster in healthy adults ≥ 50 years of age. Efficacy and persistence were minimally affected by the age of the vaccinee^46–48^. We previously showed that RZV generated higher levels of gE- and VZV-specific CD4 + Th1 memory, CD8 + cytotoxic lymphocytes, and antibodies^49–52^ than ZVL. However, adaptive T cell immune responses measured after administration of live attenuated viral vaccines typically require a week to reach their peak^53^. Thus, it is likely that innate immune responses are essential for initial control of infection with most pathogens. The ability of RZV to generate trained immunity has not been studied, but non-VZV vaccines, including live-attenuated, adjuvanted and mRNA vaccines, were shown to induce trained immunity^21,54–59^.

In the current study, we reveal VZV-specific innate immune responses in peripheral blood mononuclear cells (PBMC) after RZV administration, but not after ZVL, and then demonstrate that RZV generates durable gE-specific homologous and heterologous trained immunity in monocytes, NK, and DC that involves epigenetic modifications.

## Results

### Demographic characteristics of the study population

This study used samples from 10 ZVL and 25 RZV recipients enrolled in a randomized, double-blind study comparing the safety and immunogenicity of the two vaccines (**Supplemental Fig. 1; Supplemental Table 1**). Participants who contributed samples to this study had mean (range) ages of 67 (50–79) years. Of the 35 total participants, 18 were females and 33 were White Non-Hispanics without appreciable differences between the vaccine groups.

The frequency of activated innate and adaptive immune cells in peripheral blood increases only after RZV administration

**Figure 1:**
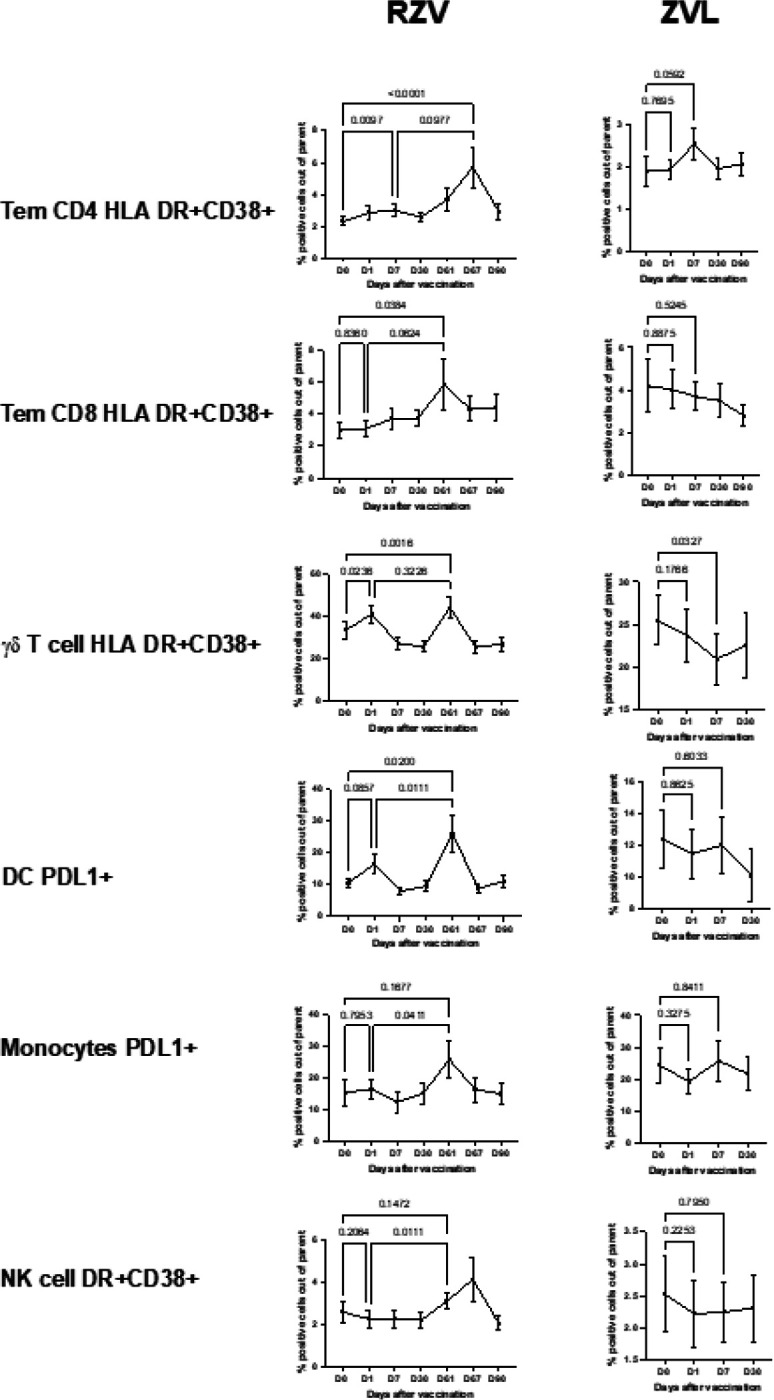
Peripheral blood innate and adaptive T cell responses to zoster vaccines. Data were derived from 10 RZV recipients who received 2 doses of vaccine on Days 0 and 60 and 10 ZVL recipients who received a single dose of vaccine on Day 0. The graphs show means and SEM of the frequencies of activated innate and T cells in peripheral blood from pre-vaccination (D0) and up to 30 days after each dose of vaccine. p values shown on the graphs for the comparison of pre- and post-vaccination responses were generated using Friedman test for repeated measures. RZV participants showed transient increases of activated immune cells in blood after each dose of vaccine. ZVL administration generated increases of activated CD4+ effector memory T cells (Tem) only.

To determine the innate immune response to zoster vaccines, we measured the activation of monocytes, DC, NK and γδ T cells in peripheral blood up to 30 days after each dose of vaccine in 10 ZVL and 10 RZV recipients. For reference, we measured activation of conventional CD4 + and CD8 + T cells and B cells representing adaptive immunity. Activation of B cells, monocytes, and DC was measured by expression of PDL1 and of the other lymphocytes by dual expression of HLA DR and CD38 (Gating strategy shown in **Supplemental Fig. 2**). We observed transient responses to vaccination in peripheral blood both in innate cells and conventional T cells but with diverging kinetics. CD4 + effector memory T cells (Tem) significantly increased after each dose of RZV with peaks on Days 7 and 67 (p ≤ 0.01; Fig. 1) and showed a trend increase between Days 7 and 67 (p = 0.098). ZVL recipients showed increases in activated CD4 + Tem on Day 7 that did not quite reach statistical significance (p = 0.06). In contrast, activated innate immune cells and CD8 + Tem increased in peripheral blood on Days 1 and/or 61 post-RZV compared to pre-vaccination levels. Activated γδ T cells reached significantly higher levels both at Days 1 and 61 post-RZV (p ≤ 0.02) compared to pre-vaccination without a significant difference between the two time points post-vaccination, while activated DC had a marginal increase on Day 1 (p = 0.09) and significant increase on Day 61 (p = 0.01) compared to pre-vaccination and significant increase from Day 1 to 61 (p = 0.02). The numbers of activated CD8 + Tems, monocytes and NK cells did not increase in the peripheral blood on Day 1 but did significantly increase on Day 61 compared to pre-vaccination or Day 1 levels. We did not detect increased activation in the peripheral blood of B cells in either vaccine group (not depicted) or in the innate cells or CD8 + Tems in the 10 ZVL recipients (Fig. 1).

Innate immune cells may be activated by cytokines generally secreted by conventional T cells—a process known as bystander activation^60^. However, differences in the kinetics of activation between CD4 + Tems and innate immune cells argue against bystander activation. Correlation analyses revealed significant associations only between activated CD4 + Tems or activated total CD4 + T cells and activated γδ T cells on Days 1 and 61 (rho ≥ 0.9 and p ≤ 0.001 by Spearman correlation analysis). The activation of other innate immune cells did not correlate with either CD4 + or CD8 + T-cell activation.

### Parallel development of trained and adaptive immune responses after RZV administration

**Figure 2:**
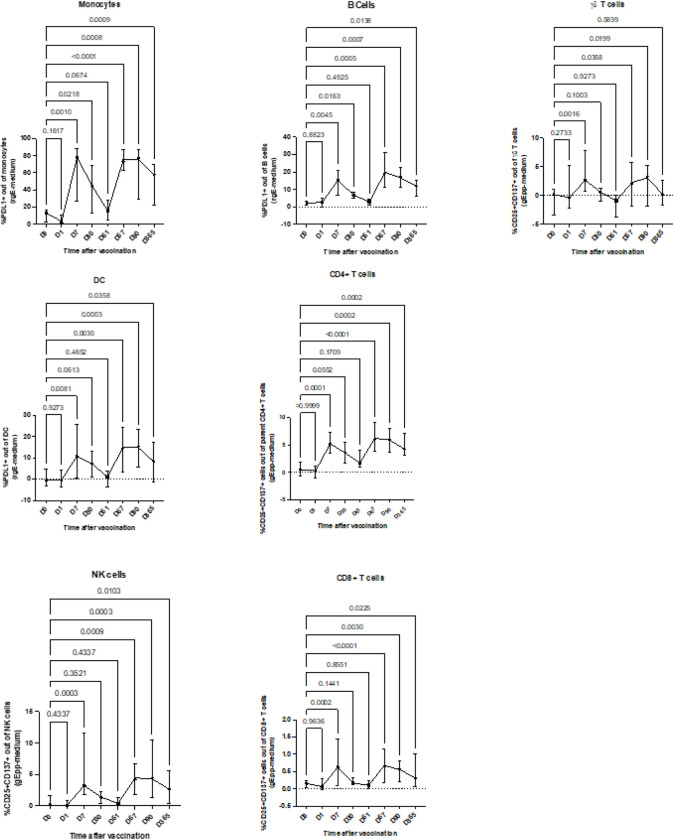
Innate and adaptive immune cell activation in response to ex vivo antigenic stimulation in RZV recipients. Data were derived from 10 participants who received 2 doses of RZV at enrollment and 60 days later. PBMC were stimulated overnight with VZV-gE peptides (gE pp) or recombinant VZV-gE (rgE) and medium control as indicated on the graphs. The graphs show mean and SEM of the frequency of activated cells in antigen-stimulated conditions after subtraction of medium control from pre-vaccination up to 1 year post-vaccination. p values for the comparison of pre-vaccination with post-vaccination results were calculated using ANOVA for repeated measures. Responses of adaptive and innate immune cells followed similar trajectories except for gd T cells. See also **Figure S4** for responses in ZVL recipients.

To test the hypothesis that RZV generates trained immunity, we stimulated ex vivo PBMCs from 10 RZV recipients with VZV-gE overlapping peptides (gE pp) or recombinant VZV-gE (rgE) and medium unstimulated controls and from 10 ZVL recipients with medium and replication competent VZV. The stimulation was performed on PBMCs obtained from all study visits in the first year after vaccination. Activation was measured by the expression of PDL1 on B cells, DCs, and monocytes and by the coexpression of CD25 and CD137 on NK cells, γδ, and conventional T cells under antigen-stimulated conditions after the subtraction of unstimulated controls (the gating strategy is shown in **Supplemental Fig. 3**). Compared with prevaccination, RZV recipients showed activation of B cells, DCs, monocytes, NKs, and conventional T cells on Day 7, which returned to prevaccination levels on Day 30, increased again on Day 67 (7 days after the 2nd dose of vaccine) and persisted above prevaccination on levels on Days 90 and 365 (Fig. 2). Activated γδ T cells also increased above prevaccination levels in response to antigenic stimulation on Days 7, 67 and 90 but returned to prevaccination levels on Day 365. ZVL recipients showed increased activation of B cells and conventional CD4 + T cells up to Day 365, while DC and conventional CD8 + T cells increased only at Days 7 and 30 (**Supplemental Fig. 4**).

Correlation analysis of the proportions of CD4 + T cells activated by VZV-gE ex vivo in RZV recipients with activated B cells, CD8 + T cells, DCs, monocytes, NK cells and γδ T cells revealed significant associations between CD4 + T cells and CD8 + T cells, γδ, and NK cells on Days 0 and 7 (**Supplemental Table 2**). Correlations between CD4 + and CD8 + T-cell activation were also noted on Days 90 and 365 (**Supplemental Table 2**). No other significant associations were noted, such that bystander activation of B cells, DCs, monocytes, γδ T cells and NK cells seemed unlikely after the 2nd dose of the vaccine.

Homologous trained immunity of DCs, monocytes and NK cells persists for several years after RZV administration

**Figure 3:**
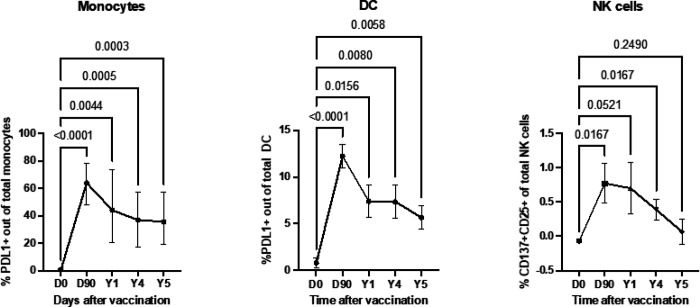
5-year persistence of innate immune responses after RZV administration. Data were derived from 14 RZV recipients who received 2 doses of vaccine at enrollment and 60 days later. The graphs show mean and SEM of the frequency of activated cells in rgE-stimulated conditions after subtraction of medium control. p values were calculated by ANOVA for repeated measures and FDR-adjusted.

To investigate the persistence of innate immune responses generated by RZV, we stimulated ex vivo PBMCs from 14 RZV recipients at 0, 3, 12, 48, and 60 months postvaccination with rgE and a medium negative control (see methods). We found that DC and monocyte responses to rgE persisted above prevaccination levels for ≥ 5 years and that NK cells persisted for 4 years (Fig. 3). In contrast, following the administration of RZV, we could not observe responses to the nonspecific inducers R848 or rhIL2 used at suboptimal doses to identify vaccine-induced increased responses (**Supplemental Fig. 5**).

### Purified monocytes and NK cells from RZV recipients exhibit homologous and heterologous trained immunity

**Figure 4:**
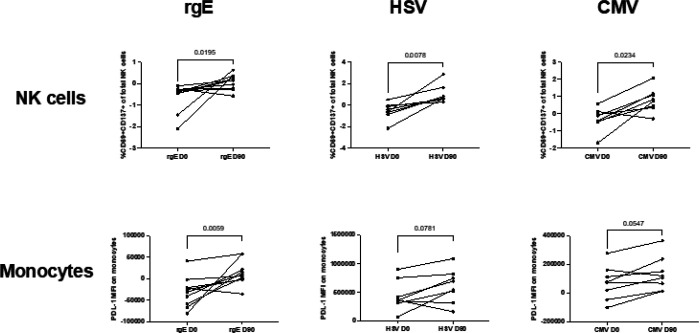
Monocyte and NK cell trained immunity after RZV administration. Data were derived from 10 RZV recipients. Monocytes and NK cells were purified using magnetic bead separation kits, combined, and incubated overnight with rgE, HSV or CMV lysate, or medium background control. Graphs show background-subtracted results before (D0) and after (D90) vaccination. p values were calculated by Wilcoxon matched-pairs signed rank test.

Monocytes and NK cells were isolated from PBMCs obtained on Days 0 and 90 from 10 RZV recipients by negative depletion using magnetic beads (see Methods section). We then combined monocytes and NK cells from the same donor at a ratio of 2:1, roughly replicating their ratios in PBMCs. The resulting cocultures had < 5% T-cell contamination. Cocultures were stimulated with rgE, CMV lysate, herpes simplex virus (HSV) lysate and medium control overnight, and activation was measured by the expression of PDL1 on monocytes and the coexpression of CD69 and CD137 on NK cells (Fig. 4). There was an increase in activation from pre- to postvaccination, both in NKs and monocytes, in response to all antigens. Notably, purified monocytes in isolation did not respond to ex vivo rgE stimulation, suggesting that cross talk between monocytes and NK cells is essential for activation.

### Epigenetic modifications associated with RZV administration

**Figure 5:**
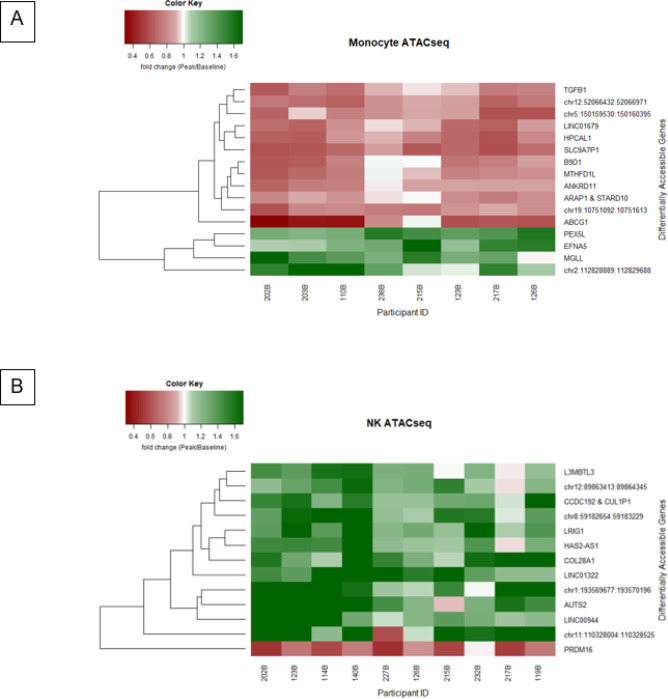
Epigenetic modifications of monocytes and NK cells obtained after RZV administration. **Panel A.** The heatmap shows the 16 genes whose accessibility significantly changed in monocytes between Days 0 and 90 after vaccination (FDR p<0.1) in 8 participants. **Panel B** The heatmap shows the 13 genes whose accessibility significantly changed in NK cells between Days 0 and 90 after vaccination (FDR p<0.1) in 10 participants.

Monocytes and other immune cells develop immunologic memory through epigenetic modifications^26^. To determine whether the monocyte memory responses to RZV were associated with epigenetic modifications, we performed bulk ATAC-seq on monocytes purified by sorting from PBMCs obtained before and 90 days after the first dose of vaccine in 8 RZV recipients. We found 16 loci with significantly modified chromatin accessibility defined by ≥ 2-fold differences in gene expression from pre- to postvaccination and an FDR-adjusted p value < 0.1 (Fig. 5A). Among the genes that may play a role in the immune response*, tgfβ1, dnam2, and arap1* exhibited decreased expression, and *mgll* and *efna5* exhibited increased expression.

ATAC-seq analysis of purified NK cells from 10 participants revealed differential expression of 13 genes from pre- to postimmunization (Fig. 5B). However, we did not identify any genes associated with immune responses.

### TGFβ1 modulates ex vivo monocyte responses to rgE in RZV recipients

**Figure 6:**
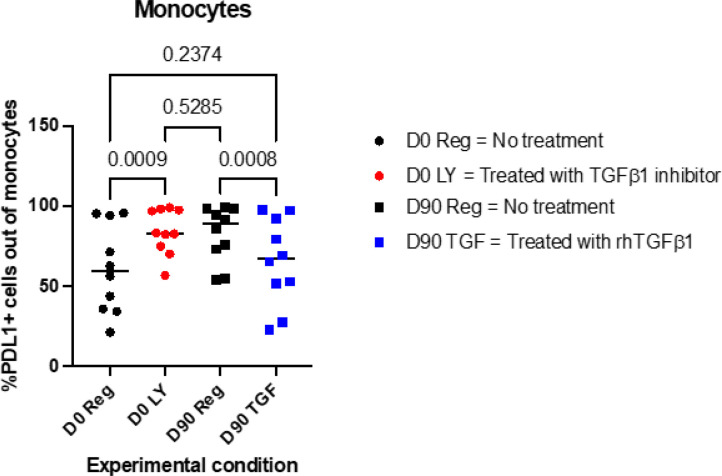
Effect of ex vivo treatment with rhTGFb1 or its inhibitor on monocyte responses to rgE stimulation. Monocytes and NK cells purified from PBMC collected from 10 RZV recipients before vaccination (D0) and 90 days post-vaccination (D90) were combined ex vivo and stimulated with rgE. A subset of D0 monocyte & NK co-cultures was also treated with the TGFb1 inhibitor LY (D0 LY), and a subset of D90 cultures were supplemented with rhTGFb1. The graph shows individual data points, means and p values calculated by ANOVA for repeated measures. LY treatment of cells collected on D0 significantly increased their activation to levels similar to D90 activation. Conversely, treatment of D90 cells with rhTGFb1 significantly decreased their activation to levels similar to D0.

We hypothesized that downregulation of the TGFβ1 pathway contributed to the trained immunity developed by monocytes in response to RZV administration. To test this hypothesis, we treated ex vivo purified monocyte and NK cell cocultures from 10 RZV recipients with rhTGFβ1 or with the compound LY3200882 (LY), which prevents the binding of TGFβ to its receptor and subsequent downstream signal transduction. Treatment of cells subjected to prevaccination with LY increased their activation in response to rgE stimulation to levels comparable to those of cells subjected to rgE-induced activation on Day 90 postvaccination (Fig. 6). Conversely, treatment of cells collected on Day 90 with rhTGFβ1 decreased rgE-induced activation to levels similar to those observed before vaccination. NK cells only partially responded to ex vivo modulation of TGFβ1 activity (**Supplemental Fig. 6**), suggesting that additional mechanisms may contribute to the development of trained immunity in NK cells.

## Discussion

The homologous trained immunity generated by vaccines may play an important role in protection against infections. For example, homologous trained immunity resulting from BCG or Ad26-SIV vectored vaccines was shown to contribute to protection against tuberculosis and simian immunodeficiency viral infections, respectively^21,54^. Trained innate immune cells may also play a role in protection against HZ^34^. Here, we show that the highly efficacious RZV generates strong innate immune responses. The activation of NK cells, γδ T cells, monocytes and DCs in the blood was detected one day after the administration of each dose of RZV. Moreover, after the 2nd dose of RZV, gE-stimulated ex vivo activation of NK cells, DCs and monocytes remained persistently greater than that before vaccination for at least 5 years, suggesting the development of trained immunity. Using their rapid response capability, innate immune cells can provide the initial response to the replication of reactivated VZV, ensuring that the reactivation of latent VZV does not progress to HZ.

The monocyte, DC and NK responses to VZV-gE ex vivo did not correlate with CD4 + T-cell responses, suggesting the development of trained immunity rather than a bystander stimulation effect. Moreover, we confirmed the development of gE-specific trained immunity in purified monocyte and NK cocultures. Notably, isolated monocytes were not differentially activated by rgE before or after vaccination. Only after the addition of purified NK cells to the purified monocyte cultures was the presence of trained immunity revealed. This observation suggested that cross talk between monocytes and other cells is essential for trained immunity generated by RZV. NK cells express CD28^61,62^, which can bind to CD80 or CD86 on the monocyte cell membrane, while monocytes express HLA Class I E^63^, which is recognized by NK cells. We propose that rgE or gE pp mediate the binding of these receptors and ligands, forming an immunologic synapse between monocytes and NK cells, which is essential for RZV-generated trained immunity.

We were also able to demonstrate monocyte and NK heterologous trained immunity against other herpesviruses, CMV and HSV, which is supported by clinical observations associating RZV administration with protection against COVID-19^64^. However, we did not observe increased heterologous responses to R848 stimulation of TLR7 and TLR8 or rhIL2. The absence of trained immunity to R848 may be explained by the divergence of intracellular pathways activated by antigens and TLR agonists. Binding of TLRs 7 and 8 elicits downstream activation of interferon regulatory factors and NFκB ^65,66^. In contrast, peptide binding to MHC activates Src kinases, and subsequent tyrosine phosphorylation is the main intracellular downstream signaling mechanism ^67^. Moreover, T-cell recognition of the peptides presented in the context of MHC on APCs creates an immunologic synapse where CD80, CD86, and CD40 APC receptors bind to their corresponding T-cell ligands, triggering additional activation signals in the APC ^68,69^ distinct from those generated by R848 binding to TLRs. We propose that cell imprinting resulting from RZV administration modulates the downstream signaling pathways triggered by peptide binding to MHC and/or costimulatory receptors on APCs but not the pathways activated by TLR7/8 stimulation or rhIL2 treatment.

Innate immune cells acquire memory through epigenetic imprinting. In the case of short-lived myeloid cells, such as monocytes and macrophages, chromatin modifications of hematopoietic stem and progenitor cells ensure the persistence of newly acquired characteristics^54,56^. For example, epigenetic features underlying trained immunity against tuberculosis and SARS-CoV-2 infection were shown to persist for 3 and 6 months after BCG administration and COVID-19 infection, respectively. Our investigation of the monocyte epigenome of RZV recipients revealed decreased accessibility of *tgfβ1, dnm2* and *arap1* and increased accessibility of *efna5* and *mgll* after vaccination.

TGFβ is a regulatory cytokine that plays a key role in the differentiation of regulatory T cells and other cell subsets. We and others have shown that TGFβ inhibits the activation and proliferation of effector T cells and the functionality of NK cells^70–72^. TGFβ also increases the differentiation of monocytes into myeloid-derived suppressor cells (MDSCs), which inhibit T-cell functionality, while neutralization or inhibition of TGFβ intracellular downstream signaling decreases differentiation into MDSCs in favor of differentiation into antigen-presenting DCs^73^. Thus, downregulation of TGFβ1 expression in monocytes after vaccination could explain the observed increase in monocyte function. Moreover, the addition of rhTGFβ1 to rgE-stimulated monocyte and NK cocultures significantly decreased monocyte and NK activation, while blocking TGFβ1 had the opposite effect. These findings support the role of TGFβ1 downregulation in establishing monocyte trained immunity.

The other genes downregulated by RZV in monocytes are involved in endocytosis and cell receptor activity^74–76^, whereas the upregulated genes play a role in cell adhesion, extravasation, and migration^77^ and in lipid metabolism and synthesis of arachidonic acid, the precursor of prostaglandins and other inflammatory mediators^78,79^. Modifications in the expression of these genes may also contribute to protective immune responses against VZV.

Although NK cells also develop epigenetic modifications after vaccination, the affected genes are not known to contribute to the immune response. CMV adaptive NK cells were previously shown to undergo epigenetic modifications through DNA methylation^32^, which was not investigated in our study. Future analyses of the effect of RZV on the DNA methylation of NK cells are planned.

DC also showed evidence of homologous trained immunity in bulk PBMC cultures stimulated with rgE. Although the bulk cultures contained gE-specific conventional T cells, we found that DC activation did not correlate with T-cell activation, suggesting that DCs were not exclusively activated by adaptive T cells. We did not pursue homologous or heterologous trained immunity in isolated DCs because they represent a very small cell population, and we did not have enough PBMCs to create isolated DCs or DC and NK cocultures, for example, to prove the development of DC trained immunity. Additional genomic assays, including multiome analyses, are planned to elucidate this aspect of DC functionality.

We observed only short-lived memory-like responses in γδ T cells after RZV. Both RZV and ZVL are associated with increased activation of CD4 + T cells and B cells, which are known to develop immunologic memory through receptor rearrangement. CD8 + T-cell activation after vaccination was observed only in RZV recipients.

Our study had limitations. Since there were no study visits between Days 1 and 7 post-vaccinations, we cannot rule out that additional increases in circulating activated innate and/or adaptive immune cells occurred between Days 1 and 7 in RZV and/or ZVL recipients. It is also possible that the relatively small number of samples tested in this proof-of-concept study might have limited our ability to detect significant increases in the innate immune responses of ZVL recipients. Moreover, prevaccination responses to VZV were greater than those to gE, making it more challenging to demonstrate postvaccination increases in response to VZV than to gE. This observation, coupled with the fact that gE-specific responses after ZVL administration are very low^50^, limits our evaluation of increases in trained immunity in ZVL recipients.

In conclusion, we showed that RZV induces trained immunity in addition to adaptive immunity, revealing a novel attribute of this vaccine. The discovery of gE-specific trained immunity elicited by RZV offers an opportunity to examine its role in protection against HZ, a disease for which a specific immune correlate with protection has not yet been identified.

## STAR Methods

Study design. This study (NCT02114333) was approved by the Colorado Multiple Institutional Review Board. All participants provided signed informed consent. The study enrolled 160 participants in good health except for those with treated chronic illnesses typical of the age of the vaccinees. All had prior varicella or resided in the USA for at least 30 years, and subsequent antibody testing by gp ELISA confirmed the presence of VZV-specific antibodies in all participants ^51^; none had prior HZ. The exclusion criteria from the study were immune suppression and recent blood products or other vaccines. The participants were divided into two age groups: ≥50–59 years (n = 46) or ≥ 70–85 years (n = 115). The older group included 70 people who received ZVL ≥ 5 years before enrollment and 44 who did not. Participants in each group were randomized at enrollment to receive one dose of ZVL followed by placebo 60 days later or RZV in two doses separated by 60 days (**Supplemental Fig. 1**). Of the 160 enrollees, 159 were vaccinated. The current study used viably cryopreserved PBMCs from blood samples collected before and up to 5 years after vaccination from participants who did not receive prior ZVL.

Flow cytometry assays. PBMCs were cryopreserved for viability within 8 h of collection and stored at ≤−150°C until use. For phenotypic characterization of innate and adaptive immune cells in peripheral blood (the gating strategy is shown in **Supplemental Fig. 2**), PBMCs were thawed, counted, and promptly stained with two different panels. Both staining panels included an initial PBS wash and viability staining with Zombie Yellow Fixability dye (BioLegend). For the APC and B-cell panels, the cells were subsequently washed with phosphate-buffered saline (PBS) supplemented with 1% bovine serum albumin (BSA; Sigma; A9576–50ML) and incubated at room temperature with Human TruStain FcX (BioLegend). Next, the cells were washed and stained for the surface markers CD14 Ax488, CD83 PE, CD141 PE-Dazzle, CD123 PerCPCy5.5, CD56 PE-Cy7, CD1c Ax700, HLA-DR APCcy7, PDL1 BV421 (BioLegend), CD3 PE-Cy7, CD19 APC (BD Biosciences), and True-Stain Monocyte Blocker (BioLegend). For the T-cell panel, the cells were washed with PBS supplemented with 1% BSA and stained for the surface markers CD16 FITC, CCR7 PE-Dazzle, CD8 PerCPcy5.5, CD56 PE-Cy7, HLA-DR APC, CD38 BV421 (BioLegend), γδ TCR PE, CD3 Ax700, and CD45RO APC-H7 (BD Biosciences). Following surface staining, both panels were washed and resuspended in PBS + 1% paraformaldehyde before acquisition on a Gallios instrument (Beckman Coulter). The data were analyzed with Flow Jo (BD Biosciences).

For responses to ex vivo stimulation (Gating Strategy in **Supplemental Fig. 3**), cryopreserved PBMCs were thawed, washed, counted, and resuspended at 5×10^6^ cells/mL in AIM-V media (Gibco; 12–055-091). VZV OKA virus, rgE (gift from GSK), gE peptides (gift from GSK), R848 (Mabtech), and rhIL2 (Sigma) were added to PBMCs at final concentrations of 60,000 plaque-forming units/ml, 5 μg/mL, 2.5 μg/mL, 0.05 μg/mL, and 95.5 ng/mL, respectively. The antigen concentrations were designed for optimal stimulation, whereas the R848 and rhIL2 concentrations were suboptimal to allow the detection of trained immunity induced by additional stimulation. After a 20-h incubation at 37°C in a CO_2_-enriched humidified atmosphere, the cells were washed with PBS, stained with Zombie yellow viability dye (BioLegend), and analyzed using the following staining panels: 1) cells in the 1-year kinetic APC panel were incubated with Human TruStain FcX and surface-stained for CD14 Ax488, CD83 PE, CD141 PE-Dazzle, CD123 PerCPCy5.5, CD56 PE-Cy7, CD1c Ax700, HLA-DR APC-Cy7, PDL1 BV421 (BioLegend), CD3 PE-Cy7, CD19 APC (BD Biosciences), and True-Stain Monocyte Blocker (BioLegend); 2) cells in the 1-year kinetics T-cell panel were surface-stained for CD16 FITC, CCR7 PE-Dazzle, CD8 PerCP-Cy5.5, CD56 PE-Cy7, CD137 APC, CD45RO BV421 (BioLegend), γδ TCR PE, CD3 Ax700, CD45RO BV421, and CD25 APC-Cy7 (BD Biosciences); and 3) cells for the 5-year kinetics were incubated with human TruStain FcX Panels 1 and 2 were acquired on a Gallios instrument, and panel 3 was acquired on a NovoCyte Quanteon instrument (Agilent). Data analysis was performed using FlowJo (BD Biosciences).

### ATAC-seq assays.

PBMCs cryopreserved before and 90 days after vaccination were thawed, washed, and counted. The cells were stained with Zombie Aqua (Biolegend), incubated with Human TruStain FcX (Biolegend), and surface-stained with CD16 FITC (Biolegend), CD14 PE-CF594 (BD Biosciences), CD56 PE-Cy7 (Biolegend), CD3 Ax700 (BD Biosciences), CD19 Ax700 (Biolegend), CD20 Ax700 (Biolegend), and HLA-DR APCH7 (BD Biosciences). The cells were then resuspended in buffer containing PBS, 1 mM EDTA (Corning), 25 mM HEPES (Corning), and 1% BSA (Sigma) and filtered before sorting on a MoFlo Astrios Cell Sorter (Beckman Coulter). Monocytes were identified as CD14 positive, HLA-DR positive, and CD3, CD19, and CD20 negative. NK cells were identified as CD14-, CD3-, CD19-, and CD20-negative and CD16- and/or CD56-positive. Before starting the ATAC-seq library prep, the cell numbers were normalized to 100,000 per sample for monocytes and 150,000 per sample for NKs. An ATAC-Seq Kit (Active Motif) was then used, and the protocol was followed according to the manufacturer’s instructions. Sequencing of the libraries was conducted with a NovaSeq 6000 (Illumina), and 40 million read pairs were recorded per sample.

### Homologous and heterologous immunity of separated monocytes and NK cells.

PBMCs cryopreserved before and 90 days after vaccination were thawed and washed before cell separation. Monocytes were enriched with the EasySep Human Monocyte Enrichment Kit (Stemcell Technologies), and NK cells were enriched with the EasySep Human NK Cell Enrichment Kit (Stemcell Technologies) according to the manufacturer’s instructions. Then, 30,000 monocytes and 15,000 NK cells were combined and cultured in AIM-V media supplemented with either rgE at 5 μg/ml or the unstimulated control. Separate prevaccination monocytes and NK cell cultures were treated with the TGFβ1 inhibitor LY2109761 (Sigma‒Aldrich) at 0.1 μg/ml, and day 90 postvaccination cocultures with recombinant human TGF-beta 1 protein (R&D Systems; 240-B-002/CF) at a concentration of 0.1 ng/ml in the presence of rgE or unstimulated control. Cultures were incubated for 48 h and stained with Zombie Aqua, PDL1 BV421 (BD Biosciences), CD69 BV786 (BioLegend), CD137 PE (BioLegend), CD16 FITC (BioLegend), CD14 PE-CF594 (BD Biosciences), CD56 PE-Cy7 (BioLegend), CD3 Ax700 (BD Biosciences), CD19 Ax700 (BioLegend), CD20 Ax700 (BioLegend), HLA-DR APCH7 (BD Biosciences), and True-Stain Monocyte Blocker. Samples were run on a NovoCyte Quanteon, and FlowJo was used for analysis.

### Statistical analysis.

ATACseq analysis: fastq files were uploaded to the Galaxy platform^80^ for analysis. FastQC was used to investigate read quality, and Cutadapt was used to cut out the adapter sequences. Reads were then mapped to the human reference genome (hg38) using Bowtie2. Uninformative reads were filtered out based on read mapping quality (< 30 on the phred quality scale), proper pairing, mitochondrial reads, and duplicate reads. The insert size distribution was then checked to verify the expected nucleosomal pattern. Files were converted from BAM to BED format, and peak calling was performed using MACS2. The bedgraph output files from the peak calling were then converted to BigWig format for easier visualization of the genome. DiffBind was used to produce a normalized read count matrix, and the matrix was read into R^81^ for differential chromatin accessibility analysis using DESeq2^82^. FDR was used to adjust for multiple comparisons, and modifications were considered significant if the p value was < 0.1. The significantly modified genes were annotated using the biomaRt package^83,^ and PathfindR^84^ was used for pathway analysis.

Flow cytometry data analysis: For the cell activation and persistence experiments, analysis of variance (ANOVA) with FDR adjustment was used to determine differences between timepoints using Prism for MacOS software (GraphPad). Purified monocyte and NK culture experiments were analyzed using the Wilcoxon matched-pairs signed rank test to identify differences in paired data before and after vaccination. ANOVA for repeated measures was used to test differences between TGFβ1 treatments, and analysis was performed using Prism software as described above.

## Figures and Tables

**KEY RESOURCES TABLE:** 

REAGENT or RESOURCE	SOURCE	IDENTIFIER
**Antibodies**		
CD14 PE-CF594	BD Biosciences	Cat# 562335, RRID:AB_11153663
CD19 APC	BD Biosciences	Cat# 555415, RRID:AB_398597
CD20 Ax700	BD Biosciences	Cat# 560631, RRID:AB_1727447
CD25 APCcy7	BD Biosciences	Cat# 557753, RRID:AB_396859
CD3 Ax700	BD Biosciences	Cat# 557943, RRID:AB_396952
CD3 PE-Cy7	BD Biosciences	Cat# 557851, RRID:AB_396896
CD45RO APC-H7	BD Biosciences	Cat# 561137, RRID:AB_10562194
CD45RO BV421	BD Biosciences	Cat# 562649, RRID:AB_2737703
γδ TCR PE	BD Biosciences	Cat# 347907, RRID:AB_400359
HLA-DR APC-H7	BD Biosciences	Cat# 561358, RRID:AB_10611876
PDL1 BV421	BD Biosciences	Cat# 568319
Brilliant Stain Buffer Plus	BD Biosciences	Cat# 566385, RRID:AB_2869761
CCR7 PE-Dazzle	BioLegend	Cat# 353235, RRID:AB_2563640
CD123 PerCPcy5.5	BioLegend	Cat# 306016, RRID:AB_2264693
CD137 APC	BioLegend	Cat# 309809, RRID:AB_830671
CD137 PE	BioLegend	Cat# 309804, RRID:AB_314783
CD14 Ax488	BioLegend	Cat# 325610, RRID:AB_830683
CD141 PE Dazzle	BioLegend	Cat# 344120, RRID:AB_2687144
CD16 FITC	BioLegend	Cat# 302006, RRID:AB_314206
CD19 Ax700	BioLegend	Cat# 363034, RRID:AB_2616936
CD1c Ax700	BioLegend	Cat# 331530, RRID:AB_2563657
CD25 BV785	BioLegend	Cat# 302637, RRID:AB_11219197
CD38 BV421	BioLegend	Cat# 356618, RRID:AB_2566231
CD56 PE-Cy7	BioLegend	Cat# 318318, RRID:AB_604107
CD8 PerCPcy5.5	BioLegend	Cat# 344710, RRID:AB_2044010
CD83 PE	BioLegend	Cat# 305308, RRID:AB_314516
HLA-DR APC	BioLegend	Cat# 361610, RRID:AB_2563200
HLA-DR APC-Cy7	BioLegend	Cat# 307618, RRID:AB_493586
CD20 Ax700	Biolegend	Cat# 302322, RRID:AB_493753
CD69 BV786	Biolegend	Cat# 310932, RRID:AB_2561370
IFNg APC	Biolegend	Cat# 506510, RRID:AB_315443
Human TruStain FcX (Fc Receptor Blocking Solution)	BioLegend	Cat# 422302, RRID:AB_2818986
True-Stain Monocyte Blocker	BioLegend	Cat# 426103
Zombie Aqua Viability Kit	BioLegend	Cat# 423102
Zombie Yellow Viability	BioLegend	Cat# 423104
**Virus Strains**		
VZV Oka	Weinberg lab	NA
**Chemicals, peptides, and recombinant proteins**		
rgE	GSK	NA
VZV-gE peptides	GSK	NA
R848	Mabtech	Cat# 3611–5X
rhIL2	Sigma	Cat# SRP3085–50UG
LY2109761	Sigma-Aldrich	Cat# SML2051
rhTGF-beta 1 protein	R&D Systems	Cat# 240–B–002/CF
penicillin/streptomyocin	Gemini	Cat# 50–753–3040
L-glutamine	Gemini	Cat# 400–106–100
EDTA	Corning	Cat# 46–034-CI
Brefeldin A	Sigma	Cat# B7651
**Other**		
30% Bovine Serum Albumen (BSA)	Sigma	Cat# A9576–50ML
AIM-V Medium	Gibco	Cat# 12–055–091
FBS	Gemini	Cat# 100–500–500
BD lysing Solution	BD Biosciences	Cat# 349202
BD Perm 2	BD Biosciences	Cat# 340973
Hepes buffer	Corning	Cat# 25–060–CI
ATAC-Seq Kit	Active Motif	Cat# 53150
EasySep Human Monocyte Enrichment Kit	Stemcell Technologies	Cat# 19059
EasySep Human NK Cell Enrichment Kit	Stemcell Technologies	Cat# 19059

## Data Availability

Raw FASTQ and data analysis files for ATAC-seq can be found at https://doi.org/10.57760/sciencedb.08957.
